# A Review on VCII Applications in Signal Conditioning for Sensors and Bioelectrical Signals: New Opportunities

**DOI:** 10.3390/s22093578

**Published:** 2022-05-08

**Authors:** Leila Safari, Gianluca Barile, Vincenzo Stornelli, Giuseppe Ferri

**Affiliations:** Department of Industrial and Information Engineering and Economics, University of L’Aquila, 67100 L’Aquila, Italy; leilasafari@yahoo.com (L.S.); gianluca.barile@univaq.it (G.B.); giuseppe.ferri@univaq.it (G.F.)

**Keywords:** sensor interface circuitry, sensor signal conditioning, Wheatstone bridge, current-mode Wheatstone bridge, SiPM, differential capacitive sensor, biomedical signal sensing, VCII, transimpedance amplifier

## Abstract

This study reviews second-generation voltage conveyor (VCII)-based read-out circuits for sensors and bioelectrical signal conditioning from existing literature. VCII is the dual circuit of a second-generation current conveyor (CCII), which provides the possibility of processing signals in the current domain while providing output signals in the voltage form. The scope of this paper is to discuss the benefits and opportunities of new VCII-based read-out circuits over traditional ones and bioelectrical signals. The achieved main benefits compared to conventional circuits are the simpler read-out circuits, producing an output signal in a voltage form that can be directly used, improved accuracy, possibility of gain adjustment using a single grounded resistor, and the possibility of connecting several SiPM sensors to the readout circuit. The circuits studied in this paper include VCII- based read-out circuits suitable for all types of sensors configured in the current-mode Wheatstone bridge (CMWB) topology, the VCII-based read-out circuits solutions reported for silicon photomultiplier, spiral-shaped ultrasonic PVDF and differential capacitive sensors, and, finally, a simple readout circuitry for sensing bioelectrical signals. There are still not many VCII-based readout circuits, and we hope that the outcome of this study will enhance this area of research and inspire new ideas.

## 1. Introduction

Nowadays, the signal conditioning of sensors and bioelectrical signals play a vital role in many areas, such as in infrastructure and environmental monitoring, healthcare, and many other applications [1,2,3,4,5,6,7]. Various types of sensors are widely used in monitoring and sensing different parameters, such as pressure, temperature, force, position, etc., thanks to the recent advances in CMOS technology, which permits integrating different types of sensors and the interface circuits into a single chip. This is definitely considered a great step toward the development of smart sensors and smart healthcare. The operations performed by the signal conditioning circuit in smart sensors and healthcare are the sensing and amplification of the sensor output/bioelectrical signal, analog-to-digital and digital-to-analog conversion, signal sampling and quantization, data processing, calibration, self-testing, and diagnosing. The first part of this system is the analog read-out circuitry, which produces an output signal dependent on the bioelectrical signals or parameters measured by the sensor. As the output of read-out circuitry is fed to the rest of the circuit for further processing, it can be viewed as the most important part due to its high impact on the overall accuracy of interface circuitry. Some of the sensor applications are categorized in the field of hand-held or portable applications, which demand low-voltage low-power operation.

Capacitive sensors, temperature sensors, pressure sensors, and silicon photo multipliers (SiPMs) are examples of the most widely used sensor types. Various approaches have been reported in the literature for the read-out circuitry of these sensors [8,9,10,11,12,13,14,15,16,17,18,19,20,21,22,23]. Traditional reported voltage-mode methods in designing read-out circuits suffer from several drawbacks. Such as high power consumption, complexity, and low-frequency operation. For example, in [17,18], read-out circuitries for differential capacitive sensors configured in the voltage-mode Wheatstone bridge (VMWB) were reported containing several different components. The complexity, large chip area, and high power consumption were their main drawbacks. Although the solution based on the current-mode approach offers circuits with less power consumption, simplicity, and higher-frequency performance, it mainly suffers from a common weakness. As most current-mode active building blocks lack a low impedance voltage output port, the existing current-mode read-out circuits do not provide an output signal in the voltage form, or they require an extra voltage buffer at output. For example, we can mention second-generation current conveyor (CCII)-based read-out circuitry as some sensor interfaces reported in [19,20].

Recently, using the duality concept, a new active building block called second-generation voltage conveyor (VCII) as the dual circuit of CCII received a boost of attention [9,11,12,13,24,25,26,27,28,29,30,31,32,33,34,35,36,37,38,39,40,41,42,43,44,45]. Similar to CCII, the operation of VCII is based on current-mode signal processing; thus, it offers all of the interesting advantages given by the CCII. Unlike CCII, VCII has a low impedance voltage output port, which offers more flexibility in applications requiring an output signal in the voltage form. Studies have been reported on VCII design and application in different areas, such as impedance simulators [30,31,32,38], filters [29,33,35,43], rectifiers [40,45], oscillators [42], etc. The reported VCII-based circuits have revealed fruitful outcomes in facing the shortcomings of traditional circuits, which have been a great motivation in utilizing VCII in the aforementioned areas. There has also been a handful of research targeting VCII-based read-out circuitries for various types of sensors and bioelectrical signals. Due to the importance of sensor and bioelectrical signal conditioning in life and healthcare today, the aim of this paper is to present a review of the research conducted on the design of VCII-based read-out circuits. We hope that this study will speed up this research area by highlighting the benefits and advantages achieved using VCII in signal-conditioning circuits. The reported VCII-based circuits for the signal conditioning of various sensors configured in current-mode Wheatstone bridge (CMWB), silicon photo multipliers (SiPMs), spiral-shaped PVDF ultrasonic sensors, differential capacitive sensors, and bioelectrical signal sensing are discussed. 

Since the VCII is a novel device, the presented work, for the first time, has the research meaning of giving a review of all of the reported read-out circuit solutions using this block. The result of this study provides an easy comparison between the old solutions and the new opportunities provided by VCII. It highlights the main achieved benefits and novelties. It helps new solutions in mitigating shortcomings of conventional solutions in designing read-out circuits. The organization of this paper is as follows. In Section 2, an introduction of VCII features and implementation is presented. In Section 3, reported VCII-based signal conditioning circuits for different types of sensors and bioelectrical signals are presented. In Section 4, comparisons and future prospects are presented. Finally, Section 5 concludes this paper.

## 2. Overview of VCII: Features and Implementation

Applying the duality concept to well-known CCII, a new active building block was found, called the second-generation voltage conveyor (VCII) [27], which was also recently compared to operational amplifiers [28].

**Figure 1:**
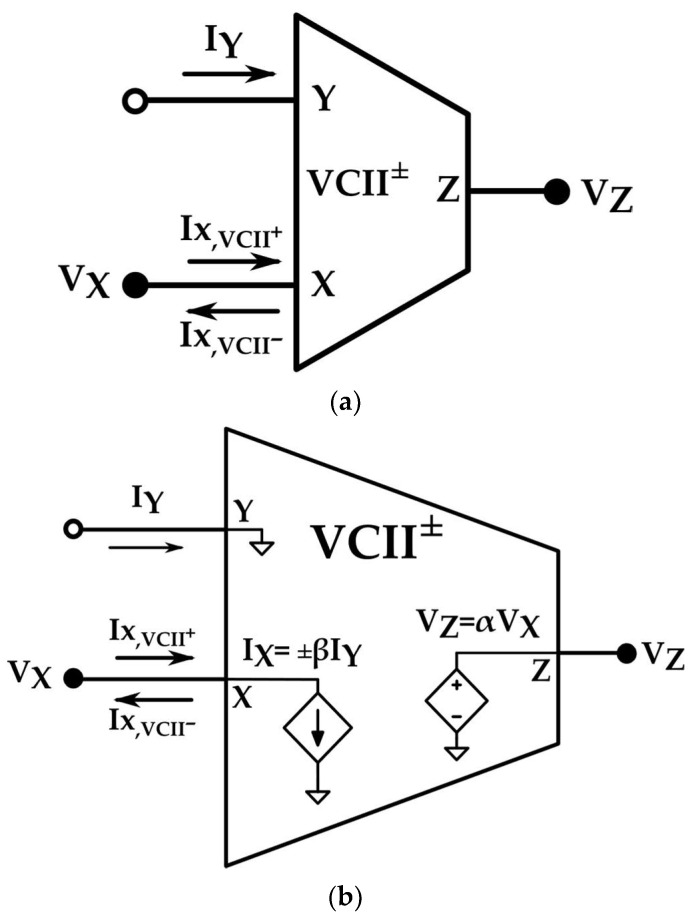
(**a**) Symbolic representation and (**b**) internal structure [27].

**Figure 2:**
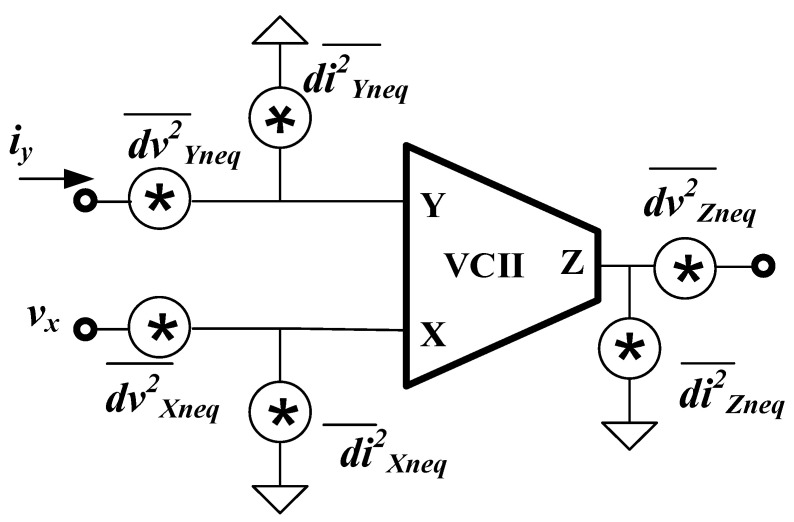
Noise model of VCII [36].

Figure 1 shows the symbol and internal structure of the VCII. According to this duality, in the VCII, there is a current buffer between the Y and X terminals, while in CCII, there is a voltage buffer between the Y and X terminals. Therefore, in the VCII, Y is a low-impedance current input port and X is a high-impedance current output port, while in CCII, Y is high-impedance voltage input port and X is low-impedance voltage output port. There is a voltage buffer between the X and Z ports of VCII, while there is a current buffer between the X and Z ports in CCII. Figure 2 shows the symbolic representation of VCII. The operation matrix of VCII is:

(1) $$\left[\begin{array}{c} I_{X}\\ V_{Z}\\ V_{Y} \end{array}\right]=\left[\begin{array}{ccc} \pm \beta & 0 & 0\\ 0 & \alpha & 0\\ 0 & 0 & 0 \end{array}\right]\left[\begin{array}{c} I_{Y}\\ V_{X}\\ I_{Z} \end{array}\right]$$

where *β* and *α* are the current gain between the Y and X ports and voltage gain between the X and Z ports, respectively, with ideal values of unity. *V_x_* and *V_z_* are the voltages at the X and Z ports, respectively. *I_Y_* and *I_X_* are the input current to the Y port and output current at the X port, respectively. For *+β*, we have VCII^+^, and for *–β*, we have VCII^−^. In addition to three-port VCII, there is another version with a five-port VCII, shown by VCII^±^, which has two X ports, two Z ports, and one Y port [38]. The extra ports provide more flexibility and freedom in some applications.

In [36], a noise model of VCII was derived, as reported in Figure 2. As shown, there are equivalent current noise and equivalent voltage noise at each port. Based on the application and port connection, some of these noise sources play important roles, while others may have a negligible effect on the circuit performance. For example, in applications where the Y port is connected to a high-impedance node, such as SiPM read-out circuits, the effect of voltage noise at the Y port ($\bar{dv_{Yneq}^{2}}$) becomes insignificant, while the equivalent current noise at the Y port i.e., $\bar{di_{Yneq}^{2}}$ must be considered in the circuit performance because it completely concerns the Y port and operates as an input signal. Therefore, for each specific application, the designer can consider the critical noise source and minimize its value to achieve the best performance. 

**Figure 3:**
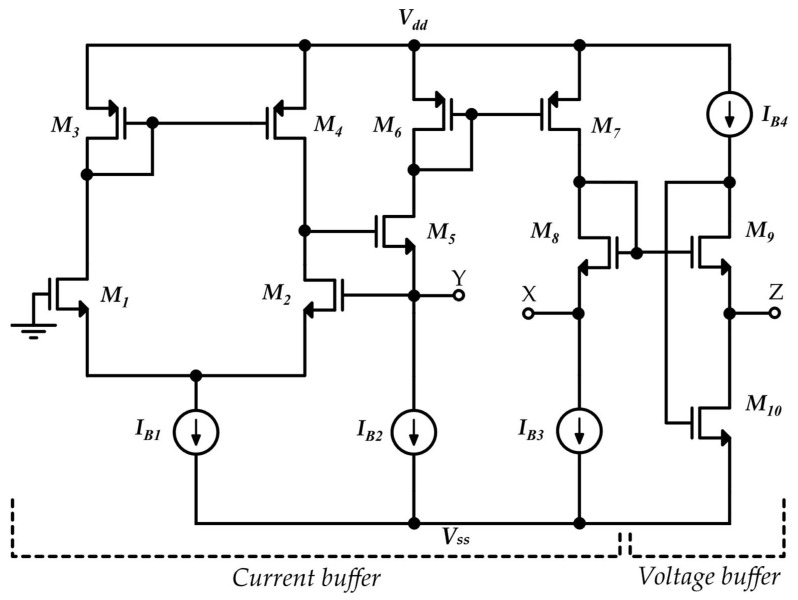
A possible simplified MOS implementation of VCII^+^ [36].

**Figure 4:**
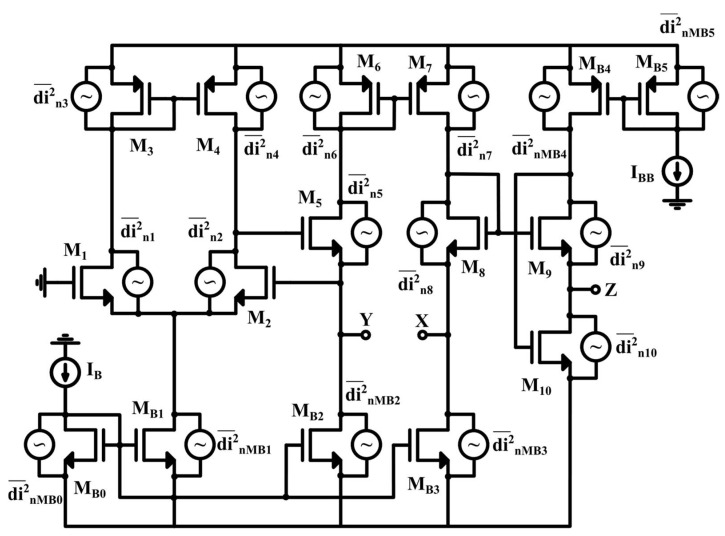
Complete VCII schematic with noise sources [36].

A basic CMOS realization of VCII^+^ is shown in Figure 3 [36], at the transistor level and in a simplified form. Here, transistors M_1_–M_7_ form the current buffer between the Y and X terminals, and transistors M_9_–M_10_ form the voltage buffer between the X and Z ports. Figure 4 shows the complete VCII schematic, also showing the equivalent output current noise produced by each transistor. The equivalent noise at each port can be achieved by analyzing the effect of each transistor’s noise. The designer can then choose the optimized size and bias current of each transistor in order to minimize its noise contribution, as explained in [36]. 

Various VCII designs have been reported. For example, a translinear-based VCII realization was presented in [34], which provides temperature-insensitive operation. In [37], a low-voltage high-drive VCII was introduced, which offers high current drive capability at X port. In [40], a rail-to-rail VCII was designed, which has a full voltage swing at the X and Z ports. 

## 3. VCII in Sensor and Bioelectrical Signal Conditioning

### 3.1. Application of VCII in Current-Mode Wheatstone Bridges

**Figure 5:**
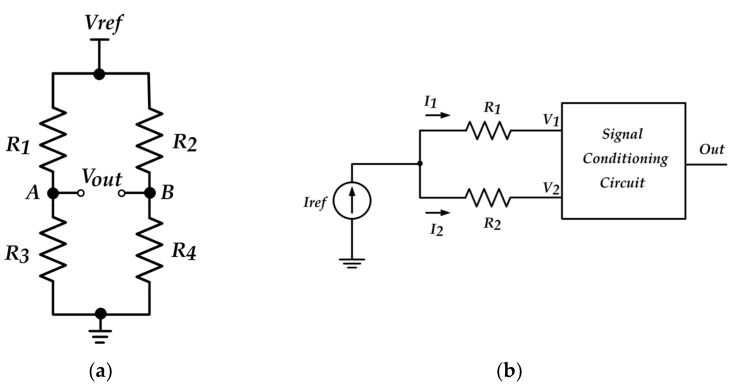
(**a**) Voltage-mode Wheatstone bridge and (**b**) current mode Wheatstone bridge [9].

The conventional voltage-mode Wheatstone bridge (VMVB) shown in Figure 5a is a network of four resistors that has wide applications in temperature, pressure, and resistive sensor signal conditioning circuits. One or some of these resistances represent sensors by the value equal to *R = R*_0_ *±* ∆*R*. A reference voltage is applied to the resistor network and an output voltage is produced in response to any change in the value of the sensor resistors. The produced output signal is processed by a voltage-handling interface circuit. Applying the duality concept, a so-called current-mode Wheatstone bridge (CMWB) was introduced based on only two resistors (Figure 5b) [8]. Compared to VMWB, the CMWB has a smaller number of resistors. In addition, the exciting signal is the current; therefore, a current-mode signal-conditioning circuit is used to process the produced signals, which enjoys the intrinsic advantages of current-mode signal processing, such as high-frequency operation. Various current-mode signal-conditioning circuits have been reported using active building blocks. such as CCII, operational floating current conveyors (OFCCs), and CDTA [8,10]. However, the reported current-mode signal-conditioning circuits suffer from some major disadvantages, such as the large number of active building blocks used in [8], which resulted in circuit complexity and high power consumption. The circuit reported in [8] consisted of three OFCCs and four resistors. Therefore, it requires high power consumption and a large chip area. The circuit reported in [10] required an extra voltage buffer at output for practical applications. In [8,10], in the case of one-sensor applications, the output signal is the non-linear function of ∆*R*. Therefore, special linearization techniques are required to produce an output signal proportional to ∆*R*. An offset canceling circuit is required to eliminate the large-value offset current, which is equal to *I_ref_/*2. In [10], the output signal is in the current form, and there is no control on gain. 

**Figure 6:**
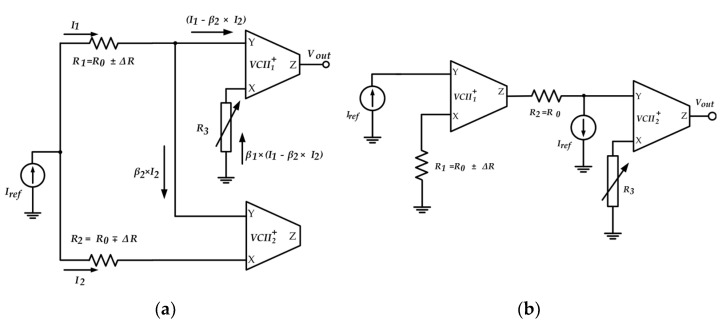
VCII-based interface circuit for CMWB [9] for (**a**) two-sensor and (**b**) one-sensor applications.

In [9], a VCII-based interface circuit for CMWB was reported, proving the high potential of VCII in eliminating the above-mentioned drawbacks. VCII-based interface circuits for both two-sensor and one-sensor applications are shown in Figure 6. In Figure 6a, *R*_1_ and *R*_2_ represent the used sensors’ equivalent circuit. Due to the very low value of parasitic resistance at the Y port of VCII, which is ideally zero, the Y ports of VCII_1_ and VCII_2_ were assumed at ground. Therefore, *I_ref_* was divided between *R_1_* and *R_2_* (producing *I_1_* and *I_2_*, respectively) based on their value. The current *I_2_*, which enters the Y port of VCII_2_, is transferred to its X port due to the current buffering action between the Y and X ports with the gain of *β_2_* with a value close to unity, producing *β*_2_*I*_2_ at the X port of VCII_2_. A current subtraction between I_1_ and β_2_I_2_ is performed at the Y port of VCII1. The resulting current enters the Y port of VCII1, which is then transferred to its X port by gain of *β*_1_ (the current gain between the Y and X ports of VCII1), where it is converted to the proportional voltage by *R*_3_. Due to the voltage-buffering action between the X and Z ports, the produced voltage is transferred to the Z port of VCII1 with the gain of *α*_1_ (the voltage gain between the X and Z ports of VCII_1_). To follow the given explanations, the related current and voltage signals are shown in Figure 6a. In the case of a single sensor, which is shown in Figure 6b, *R*_1_ is the used sensor and *R*_2_ is a resistor with a value equal to *R*_0_ of the used sensor. Here, *I_ref_* enters the Y port of VCII1, which is transferred to its X port and converted to voltage by *R*_1_. The produced voltage is transferred to VCII_1_’s Z port by the gain of *α*_1_, where it is converted to a current by *R*_2_. The current subtraction performed at the Y port of VCII_2_ removes the DC part of the current signal entering the Y port of VCII_2_. The gain-controlling resistor *R_3_* produces an output signal proportional to ∆*R*. The circuits are intrinsically linear for both two-sensor (Equation (2)) and one-sensor (Equation (3)) cases:

(2) $$V_{out}\approx \frac{\pm \Delta R}{R_{0}}\alpha_{1}R_{3}I_{ref}$$

(3) $$V_{out}\approx \frac{\pm \Delta R}{R_{0}}\alpha_{2}\beta_{2}R_{3}I_{ref}$$

The first advantage of VCII-based signal-conditioning circuits is that the output signal is in the voltage form produced at the low impedance Z port of VCII. Therefore, for practical applications, no extra voltage buffer is required. Fortunately, the need for offset cancelation circuits is also alleviated here. A simple KCL analysis shows that the DC offset current is intrinsically eliminated at the Y port of VCII1 in Figure 6a. In Figure 6b, the offset current is simply eliminated by adding I_ref_ to the Y port of VCII2, where, for *i* = 1,2, *α_i_* and *β_i_* are the voltage gain and current gain of the ith VCII, respectively, with both values close to unity. The conditions *α*_1_ *≈* 1 and *β*_2_*α*_2_ *≈* 1 must be satisfied for Equation (2) and Equation (3), respectively. Fortunately, as the values of *α* and *β* are very close to unity, these conditions are usually met. The gain of circuit can also be simply adjusted by the value of *R*_3_. In [9], using an electronically variable resistor for *R*_3_, the gain is electronically varied using a control voltage.

### 3.2. Application of VCII in Silicon Photo Multipliers

**Figure 7:**
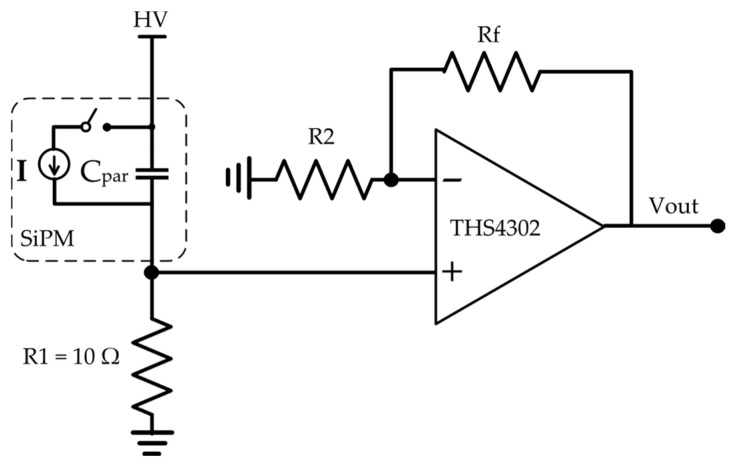
Conventional OA-based VA as readout circuitry for SiPM [14].

Recently, large-current-gain silicon photo multipliers (SiPM) have made them the best choice for photo sensors [11,12,13,14,15]. It may seem that measuring the incident photons using large-gain SiPMs is easy. However, the main challenge in designing an efficient read-out circuitry for SiPMs is dealing with their large output capacitance (*C_par_*). In particular, for an array of *N* SiPMs connected in parallel, the output capacitance becomes even larger (*C_ToT_ = NC_par_*), with values up to thousands of pF. On the other hand, the SiPM read-out circuitry must fulfill other requirements, such as a fast response time, high linearity, low added noise, and sufficient gain. These features are mandatory for the proper acquisition of incoming signals. To mitigate the effect of large input capacitance, low input impedance is required for read-out circuitry. The conventional methods of designing SiPM read-out circuitry are common-gate (CG)–common-base (CB) amplifiers, operational amplifier-based voltage amplifiers (VAs), and operational amplifier-based transimpedance amplifiers (TIAs) [14]. All of these solutions provide low input impedance to reduce the effect of the large parasitic capacitance of SiPMs. Unfortunately, the CG and CB amplifiers suffer from inappropriate output impedance. In fact, they require an extra voltage buffer at output. In addition, the gain-dependent bandwidth of OA-based VAs and TIMAs makes these structures unattractive. Let us consider OA-based VAs in more detail, which is shown in Figure 7 [14].

Here, the current signal from SiPM is converted to voltage by *R*_1_ at first. The produced voltage is amplified by the OA configured in a negative feedback loop. The main weakness of this circuit is its constant-gain bandwidth product. Therefore, by increasing the gain value, the bandwidth reduces. On the other hand, the value of *R*_1_ must be small enough to reduce the effect of *C_par_*. This indicates that the produced input voltage is very small and very prone to the input noise of OA.

**Figure 8:**
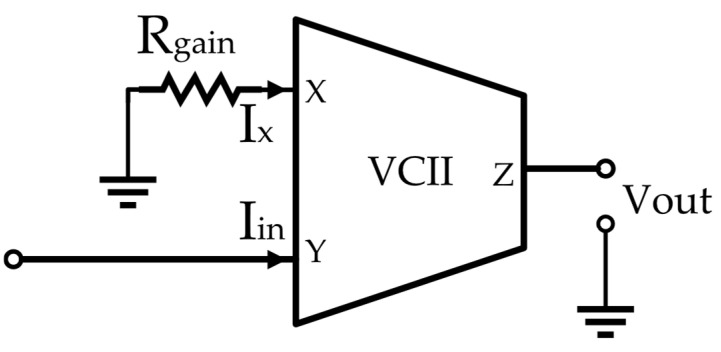
VCII as a transimpedance amplifier [11,12,13].

A helpful solution in providing high-frequency performance is using the current-mode signal-processing technique. For minimum possible additive noise, the straightforward solution is adopting a very simple structure with a smaller number of components, having low input impedance at the Y port and a very simple internal structure that includes only a simple current buffer, and the voltage buffer makes VCII a very suitable candidate for SiPM read-out applications. In addition, as signal processing in VCII is performed in the current domain, a fast response time and high frequency performance are ensured. Interestingly, as shown in Figure 8 [11,12,13], by connecting the X port to a resistor *R*_*g*_, VCII operates as a transimpedance amplifier between the Y and Z nodes with gain equal to:

(4) $$\frac{V_{out}}{I_{in}}=\alpha \beta R_{gain}$$

where *α* and *β* are the voltage gain and current gain of VCII. For Equation (4), we must have *R_g_* << *r_X_*. *I_in_* is the input signal to the circuit and *V_out_* is the produced output signal. The value of *r_X_* is usually larger than 100 kΩ, as reported in [29,30,31,32,34,35,42,43,44]. Therefore, by adopting the value of *R_gain_* at 10 kΩ, large values of gain up to 80 dB are achievable. Importantly, the achieved gain is independent of bandwidth because the VCII is not configured in a negative feedback loop. The incoming input current signal is detected and converted to a proportional voltage signal, which is available at the low-impedance Z port of VCII. Therefore, the output signal can be directly used without any need for extra voltage buffers.

**Figure 9:**
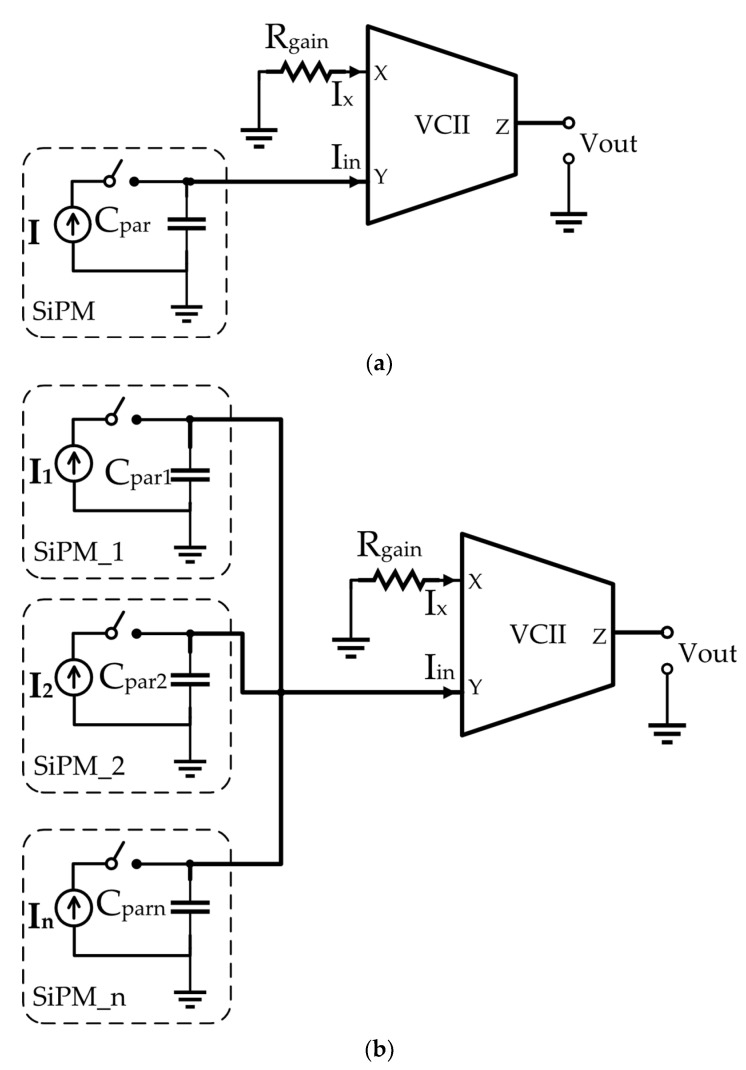
VCII-based readout circuitry for (**a**) a single SiPM and (**b**) an array of *n* SiPMs [13].

Figure 9a,b show the SiPM read-out circuitry for a single SiPM and an array of *n* SiPMs. In Figure 9a, C_par_ is the parasitic capacitance and I_1_ is the output current of the SiPM sensor, respectively. In Figure 9b, for *i* = 1 − n, C_par,i_ and *I_i_* are the practice capacitance and output current of the nth SiPM, respectively. In Figure 9b, by connecting the *i*th switch, the related SiPM is connected to the VCII-based transimpedance amplifier Y node. Then, the sensors’ output current is converted to the proportional voltage by VCII. A comprehensive study on the VCII internal noise reduction and optimization techniques was reported in [36], which must be considered in the design of VCII’s internal structure intended to be used in SiPM interface circuitry.

### 3.3. Application of VCII in an Ultrasonic PVDF Interface Circuit

Piezoelectric sensors are widely used for the generation and reception of ultrasounds in different fields, such as echolocation and communication systems, medical treatment, etc. As an example, in [25], the VCII-based trans-impedance configuration reported in Figure 6 was used as the first solution for the interface circuitry of ultrasonic PVDF sensors. The low impedance at the Y port of VCII allowed directly connecting the spiral-shaped PVDF sensor to a preamplifier. Traditionally, OAs are used as preamplifiers in ultrasonic PVDF sensors, which are constrained by the gain-dependent bandwidth, high complexity, high power consumption, etc. The second solution for ultrasonic PVDF sensors proposed in [25] shown in Figure 8 is a configurable VCII-based low-pass or band-pass filter which performs a filtering action on the incoming signal. Measurement results obtained using a discrete prototype are also reported in [25].

The transfer function between *I_in_* and *V_out_* is:

(5) $$\frac{V_{out}}{I_{in}}=\frac{Z_{2}Z_{3}Z_{4}}{\left(Z_{1}+Z_{2}\right)\left(Z_{3}+Z_{4}\right)}$$

For *Z*_1_ = 1*/sC*_1_, *Z*_2_ = *R*_2_, *Z*_3_ = 1*/sC_3_*, and *Z*_4_ = *R*_4_, Equation (5) transforms into a second order bandpass transfer function as:

(6) $$\frac{V_{out}}{I_{in}}=\frac{sC_{1}R_{2}R_{4}}{1+s\left(C_{1}R_{2}+C_{3}R_{4}\right)+s^{2}C_{1}C_{3}R_{2}R_{4}}$$

For *Z*_1_ = *R*_1_, *Z*_2_ = 1*/sC*_2_, *Z*_3_ = *R*_3_, and *Z*_4_ = 1*/sC*_4_, Equation (5) is a second-order low-pass transfer function:

(7) $$\frac{V_{out}}{I_{in}}=\frac{sC_{1}R_{2}R_{4}}{1+s\left(C_{2}R_{1}+C_{4}R_{3}\right)+s^{2}C_{2}C_{4}R_{1}R_{3}}$$

**Figure 10:**
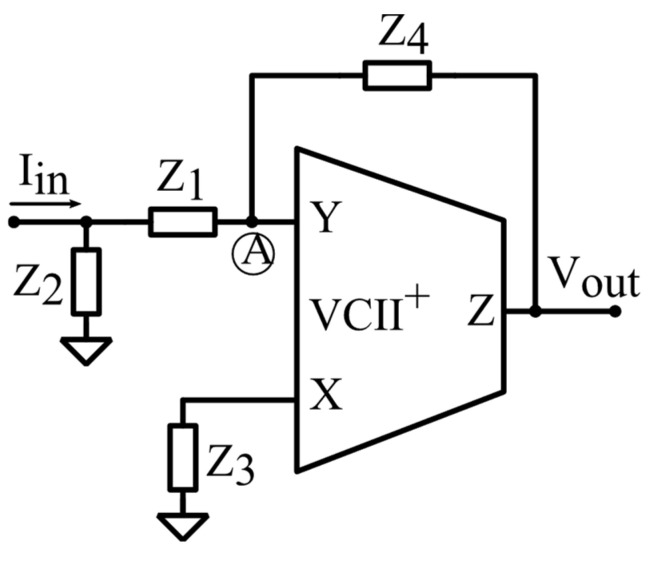
VCII-based reconfigurable low-pass band-pass filter for ultrasonic PVDF sensors [25].

Therefore, using the circuit in Figure 10, the noise associated with the input signal is eliminated by choosing the appropriate filter function and the purified input signal is transferred to an appropriate voltage single output, which is available at the Z port of the VCII. 

### 3.4. Application of VCII in Differential Capacitive Sensors

Capacitive sensors are an essential part of many sensing systems, such as accelerometers, pressure sensors, position sensors, etc. [16,17,18,19,20,21,22,23]. Differential capacitive sensors intrinsically mitigate the effect of unwanted common-mode signals and parasitic effects; therefore, they make it possible to use low-cost and simple read-out circuitry. The difference in capacitance is converted to voltage, frequency, or digital output by read-out circuitry. The conventional read-out circuits for differential capacitive sensors suffer from extra complexity, which fails to fulfill the easy integration, low power consumption, and low chip area requirements. For example, in [16,17,18,19,20,21,22,23,24], bridge-based read-out circuitry was based on the modulation–demodulation technique, which consists of various blocks as the multiplier, differential amplifier, PI controller, and filter. To be more specific, the used differential amplifier itself consists of three OTAs and six high large-value resistors, in addition to a separate reference voltage. For proper operation, strict matching between six resistors is mandatory. The large requirement for chip area and high power consumption are the main problems of this solution. In the conventional solutions reported in [16,17,18,19,20,21,22,23], a large number of switches are employed requiring additional controlling clock signals. This solution suffers from limited achievable accuracy due to the problems caused by the clock feedthrough and charge injection errors of switches.

**Figure 11:**
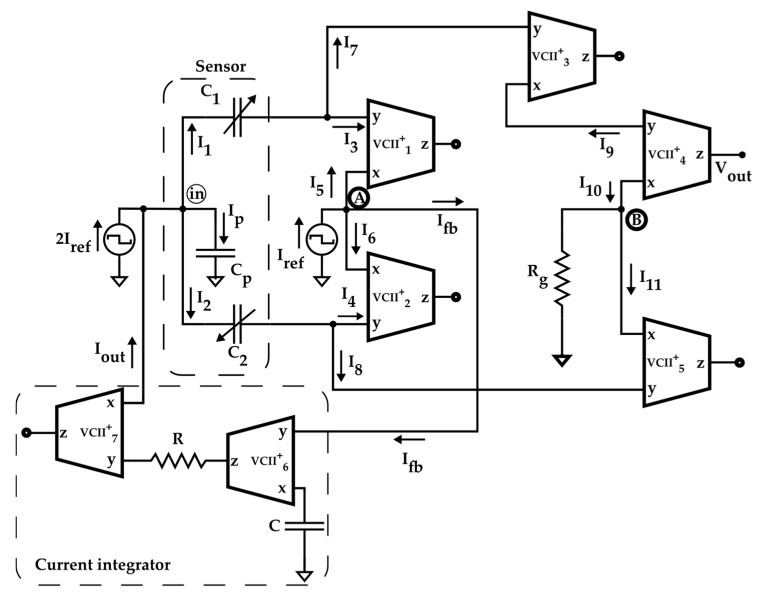
VCII-based readout circuit for differential capacitive sensors [24].

In [24], for the first time, read-out circuitry for differential capacitive sensors using VCII is reported. The circuit is shown in Figure 11. It operates based on capacitance to voltage conversion. C_p_ is the parasitic capacitance associated with the sensor. The sensor’s capacitors C_1_ and C_2_ are excited by a square-wave current signal; therefore, they are automatically charged and discharged without any need for switches. The circuit is designed in a way that the current wasted by C_p_ is measured and compensated. The used VCII_1_-VCII_2_ forms a current summation/subtraction. Therefore, the sum of the currents at C_1_ and C_2_ is produced at node *A*, where is it subtracted from *I_ref_*. By this, the amount of current stolen by C_p_ is produced as *I_fb_*, which is fed back to the input node by the VCII-based current integrator composed of VCII_6_-VCII_7_. After compensating for the effect of C_p_, the sensor’s current is subtracted at node B by VCII_4_-VCII_5_. VCII_3_ is used to invert the C_1_ current needed for current subtraction. The resulting signal is converted to a proportional voltage by *R_g_*, which is transferred to the output node by VCII_4_. 

The operation is completed by only seven VCIIs, two low-value resistors, and one capacitor. The main feature of this solution is that signal processing is completely performed in the current domain, granting the read-out circuitry a fast response time. In addition, using only one type of active building block, the circuit enjoys extreme simplicity and very easy integration requiring a low chip area because each VCII is composed of only 10 MOS transistors, and the used resistors are of low values. The other distinguishing feature is that the effect of parasitic capacitance is effectively reduced using a simple VCII-based integrator in the negative feedback loop. To provide high accuracy and avoid the problems caused by switches, the sensors are excited by a square-wave signal. 

The simulation and experimental results reported in [24] prove the high potential of VCII for use in low-cost, highly accurate, and fully integrated read-out circuitry for differential capacitive sensors. In the traditional method reported in [23], parasitic capacitance is compensated using three instrumentation amplifiers, an integrator, low-pass filter, and a voltage-controlled negative impedance convertor. Comparing the solution in [23] and the VCII-based method of [24], we find that the VCII offers a much simpler solution compared to previous methods using conventional building blocks.

### 3.5. Application of VCII in Biomedical Sensors

**Figure 12:**
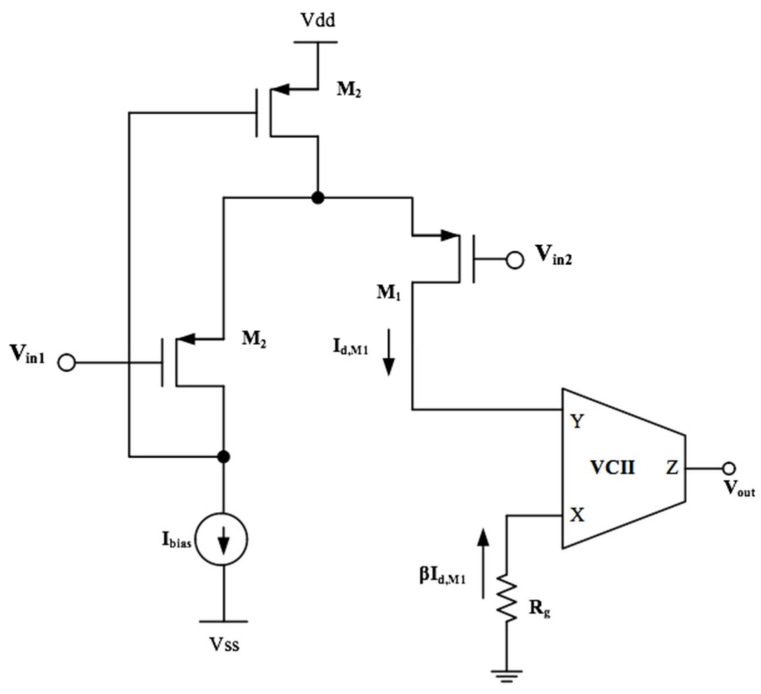
VCII-based readout circuit for bioelectrical sensing [26].

A general electrical biosignal is characterized by a low amplitude value of up to 1 mV and frequency of 0.5 Hz–10 kHz [26,46]. The challenging part is that this weak signal is accompanied by a large noise signal. For the read-out circuitry, it is required to detect and distinguish the low-value signals from unwanted noise. For portable applications, low power consumption and a small size are also fundamental features. In [26], a read-out circuit was reported using a fully differential transconductance amplifier, two pseudo resistors, two switches, four capacitors, and a standard instrumentation amplifier (IA), including a differential amplifier and three resistors. Evidently, the matching between resistors in IA highly affected the overall accuracy of the read-out circuit. The difficulty in integration is the direct result of the large number of used components. Fortunately, in this area, VCII provides a very simple solution. The results of the study reported in [26] reveal a very simple and effective VCII-based read-out circuit for biosignals. The circuit, shown in Figure 12, employs a differential floating voltage flower (FVF) (formed by *M*_1_*–M*_3_), a VCII, and a single grounded resistor *R_g_*. Two small-size capacitors are also used at the inputs to block the DC signals. Analysis of this circuit shows that the drain current of *M_1_* is expressed as:

(8) $$I_{d,M1}=\frac{1}{2}\mu_{p}C_{ox,p}{\left(\frac{W}{L}\right)}_{M1,M2}{\left(V_{in1}-V_{in2}\right)}^{2}$$

Due to the existence of a current buffer between the Y and X terminals of VCII, *I_d,M_*_1_ is transferred to the X terminal, which is terminated to resistor *R_g_*. A voltage proportional to *I_d,M_*_1_ is produced at the X terminal, which is copied to the Z terminal by means of the internal voltage buffer between the X and Z terminals. The produced output voltage is:

(9) $$V_{z}=V_{out}=-\alpha \beta R_{g}I_{d,M1}=-\frac{1}{2}\alpha \beta R_{g}\mu_{p}C_{ox,p}{\left(\frac{W}{L}\right)}_{M1}{\left(V_{in1}-V_{in2}\right)}^{2}$$

The resistance *R_g_* acts as a gain-controlling resistor. In [24], using an electronically tunable resistor for *R_g_*, the possibility of electronically tunable gain was also provided. A total number of 27 MOS transistors was used, indicating a very low required chip area with overall power consumption of only 20 μW. 

## 4. Comparison and Future Prospects

In this section, some comparison tables between VCII-based read-out circuits and conventional ones are reported. Starting from Table 1, due to the processing signals in the current domain, the circuit described in [24] achieves comparable or better performances with respect to the other voltage-mode counterparts with a much simpler topology and the need for only one type of active building block. Moreover, if compared to [47], which also processes signals in the current domain, the presence of VCIIs enables designers to disregard switching structures, therefore neglecting the need for clock signals. Table 2 compares VCII-based SiPM interfaces [13] to other available solutions. It is evident that the VCII-based solution allows achieving very high transimpedance gain with the lowest power consumption and an acceptable bandwidth due to the peculiar feature of the VCII that the bandwidth remains constant, regardless of the gain of the amplifier. In addition, the low-impedance current input port allows VCII-based circuits to perform the current summation function very easily. This property is very useful in SiPM read-out circuits to add the required number of sensors to VCII Y nodes while reducing the effect of the parasitic capacitance of the SiPM sensors. In the case of read-out circuitry for CMWBs, the comparison is shown in Table 3. The current summation property of the VCII allows reducing the DC component of the output signal and provides intrinsic linearity for the single-sensor case. Table 4 summarizes the benefits of using VCII read-out stages for ultrasonic sensor applications: as shown, it is possible to achieve a very high gain together with a very large bandwidth [25], enabling the designer to take advantage of the novel and wideband shapes of the ultrasonic transducer. Lastly, Table 5 reports the application of the VCII to implement a biosignal interface circuit [26], comparing it with other techniques available in the literature. It is possible to achieve good power consumption while being able to continuously tune the gain of the amplifier stage. Moreover, the input impedance of the interface can be easily designed to be higher than the GΩ in the required frequency band. 

Although VCII is not yet available as IC, in the measurement results reported so far, it has been implemented simply using two AD844s. However, if VCII is available as a custom integrated circuit chip, better accuracy results, and lower power consumption can be achieved.

## 5. Conclusions

In this paper, VCII-based solutions for read-out circuits of various types of sensors and bioelectrical signals are reviewed and compared with conventional solutions. With respect to other active building blocks, VCII is more flexible due to having a low-impedance current-input Y port, which makes current summation easy, a low-impedance voltage-output Z port, and processing signals in the current domain. The results of this study show that the following advantages are achieved for different VCII-based read-out circuits: 1—an intrinsically linear very simple read-out circuit for sensors configured in the CMWB configuration with gain-controlling opportunity using a grounded resistor; 2—an improved accuracy with parasitic-insensitive operation for differential capacitive sensor read-out circuitry; 3—a very simple readout circuitry for SiPM sensors with reduced sensitivity to large parasitic capacitance associated with the sensor; 4—a very simple and low-power read-out circuitry for bioelectrical and PVDF sensors. More importantly, in all reported read-out circuits, the produced output signal is in the voltage form, and is available at the low-impedance Z port of the VCII, which can be also cascaded to other circuits directly. 

## Figures and Tables

**Table 1:** Comparison between a VCII-based read-out circuit for differential capacitive sensors and conventional ones.

Ref.	[18]	[21]	[22]	[24] *	[47]	[48]	[49]
Approach	C-V	C-V	C-Digital	Mixed	C-I	C-V	C-V
Variation range	±100%	±50%	±50%	±100%	±100%	±60%	−30%–100%
C_bl_	140 pF–14 nF	500 pF	400 pF	10–200 pF	1 pF	20 pF	400 pF
Linearity error	0.5–0.8%	<0.03%	<0.2%	<1.9%/<0.9%	±1.5%	<0.1%	<0.45%
Sensitivity	71 mV/pF	5 mV/pF	4 counts/pF	412/21 mV/pF	50 nA/fF	833 mV/pF	Non linear
Typology	Discrete	Discrete	Discrete	Discrete	Integrated	Discrete	Discrete

* VCII-based circuit.

**Table 2:** Comparison between a VCII-based SiPM read-out circuit and conventional ones.

Ref.	Tech.	Supply	Power	T-I Gain	BW	Noise
[13] *	CMOS 130 nm	1.2 V	0.34 µW	100 dB	10 MHz	27 mV_rms_ (output)
[50]	CMOS 350 nm	3.3 V	0.68 µW	100 dB	50 MHz	1300 e-(ENC)
[51]	CMOS 350 nm	3.3 V	0.68 µW	500	150 MHz	2 µV_rms_ (input)
[52]	CMOS 350 nm	3.3 V	0.68 µW	/	/	6.9 mV_rms_ (output)
[53]	SiGe 130 nm	−3.2 V	82 µW	56 dB	45 GHz	30.6 pA/√Hz

* VCII-based circuit.

**Table 3:** Comparison between a VCII-based CMWB read-out circuit and conventional ones.

Ref.	Active Building Block	#of Active Building Block	#of Resistors	Intrinsic Linearity for One Sensor Case	Output Signal
[8]	CDTA	1	0	No	Current
[9] *	VCII	2	1	Yes	Voltage
[10]	OFCC	3	5	No	Current

* VCII-based circuit.

**Table 4:** Comparison between a VCII-based PVDF sensor read-out circuit and conventional ones.

Sensor	Active Device	Number of Processing Stages	Filtering Stage	Gain	BW (KHz)	Power Consumption (mA)
Cylindrical 40 KHz	MOS stage	3	Bandpass	31 dB	100	30
Cylindrical 80 KHz	Op-Amp stage	3	Bandpass	61 dB	67	12 (estimated)
[25] *	VCII	1	None	86 dBΩ	>103	6

* VCII-based circuit.

**Table 5:** Comparison between a VCII-based read-out circuit for biosignal conditioning and conventional ones.

Parameter	2020 [26] *	2016 [54]	2018 [55]	2019 [56]	2019 [57]	2018 [58]
CMOS Technology	LFoundry 150 nm	180 nm	180 nm	180 nm	180 nm	500 nm
Supply voltage	±0.6 V	1.2 V	1 V	1.2 V	1.2 V	3.3 V
Static power consumption	20 µW	0.9 µW	0.25 µW	8.1 µW	2.48/5.46 µW (AP/LFP)	28.05 µW
Amplifier gain (dB)	0–33 (continuous Tuning)	30/50	25.6	26/32/35.6 (Selectable)	40/20 (AP/LFP)	49.5 (Untunable)
f_HPF_ (Hz)	10^−5^	6.3	4	0.025/0.25/0.5/1.5/32/65/125/260	-	13
f_LPF_ (kHz)	174~3980	0.175	10	1/11.4/125	100/1000 (LFP/AP)	9.8
Z_in_	3.2 GΩ (@10 kHz)	20 MΩ	200 MΩ @100 Hz	-	-	-
Z_out_	1.2 kΩ (@10 kHz*)*	-	-	-	-	-
THD @frequency reference	1.02% (−39.8 dB) @Vin = 2 mVpp, Vctrl = 0 V, 10 kHz)	0.4%@1 mVpp 10 Hz	-	-	-	1% @ 0.7 mVpp, 10 kHz
Noise voltage (input referred)	5.4 µV_RMS_ (0.1 Hz~10 kHz)	2.6 µVRMS (0.5 Hz~400 Hz)	3.32 µV_RMS_ (250 Hz~10 kHz)	6.75 µV_RMS_ (0.5~11.4 k, 40 dB)	AP: 3.44 (0.25 k~10 k) LFP: 6.88 (0.025~600)	1.88 µV_RMS_ (0.03 Hz~11 kHz)
NEF	8.3	6.6	1.07	7.29	NA	2.3

* VCII-based circuit.

## Data Availability

Not applicable.
